# Raising awareness of antimicrobial resistance: development of an ‘antibiotic footprint calculator’

**DOI:** 10.1093/jac/dkad113

**Published:** 2023-04-18

**Authors:** Ravikanya Prapharsavat, Rattanasiri Kittikongnapang, Chokdee Smithkittipol, Warute Udomrat, Sukanya Numsawat, Anastasia Hernandez-Koutoucheva, Bhensri Naemiratch, Chalita Chomkatekaew, Chanaki Amaratunga, John Bleho, Kalai Mathee, Mira Leonie Schneiders, Napat Khirikoekkong, Nipaphan Kanthawang, Nithima Sumpradit, Rapee Suveeranont, Supa-at Asarath, Tassawan Poomchaichote, William Schilling, Xin Hui S Chan, Anne Osterrieder, Phaik Yeong Cheah, Direk Limmathurotsakul

**Affiliations:** Mahidol Oxford Tropical Medicine Research Unit, Mahidol University, Bangkok, 10400, Thailand; Greenpeace Thailand, Bangkok, Thailand; World Animal Protection, Bangkok, Thailand; PunchUp World, Bangkok, Thailand; Food and Drug Administration, Ministry of Public Health, Nonthaburi, Thailand; Centre for Tropical Medicine and Global Health, Nuffield Department of Medicine, University of Oxford, Oxford, UK; Mahidol Oxford Tropical Medicine Research Unit, Mahidol University, Bangkok, 10400, Thailand; Mahidol Oxford Tropical Medicine Research Unit, Mahidol University, Bangkok, 10400, Thailand; Mahidol Oxford Tropical Medicine Research Unit, Mahidol University, Bangkok, 10400, Thailand; Centre for Tropical Medicine and Global Health, Nuffield Department of Medicine, University of Oxford, Oxford, UK; Mahidol Oxford Tropical Medicine Research Unit, Mahidol University, Bangkok, 10400, Thailand; Department of Human and Molecular Genetics, Herbert Wertheim College of Medicine, Florida International University, Miami, FL, USA; Biomolecular Sciences Institute, Florida International University, Miami, FL, USA; Centre for Tropical Medicine and Global Health, Nuffield Department of Medicine, University of Oxford, Oxford, UK; Mahidol Oxford Tropical Medicine Research Unit, Mahidol University, Bangkok, 10400, Thailand; Mahidol Oxford Tropical Medicine Research Unit, Mahidol University, Bangkok, 10400, Thailand; Food and Drug Administration, Ministry of Public Health, Nonthaburi, Thailand; PunchUp World, Bangkok, Thailand; Mahidol Oxford Tropical Medicine Research Unit, Mahidol University, Bangkok, 10400, Thailand; Mahidol Oxford Tropical Medicine Research Unit, Mahidol University, Bangkok, 10400, Thailand; Mahidol Oxford Tropical Medicine Research Unit, Mahidol University, Bangkok, 10400, Thailand; Centre for Tropical Medicine and Global Health, Nuffield Department of Medicine, University of Oxford, Oxford, UK; Centre for Tropical Medicine and Global Health, Nuffield Department of Medicine, University of Oxford, Oxford, UK; Mahidol Oxford Tropical Medicine Research Unit, Mahidol University, Bangkok, 10400, Thailand; Centre for Tropical Medicine and Global Health, Nuffield Department of Medicine, University of Oxford, Oxford, UK; Mahidol Oxford Tropical Medicine Research Unit, Mahidol University, Bangkok, 10400, Thailand; Centre for Tropical Medicine and Global Health, Nuffield Department of Medicine, University of Oxford, Oxford, UK; Mahidol Oxford Tropical Medicine Research Unit, Mahidol University, Bangkok, 10400, Thailand; Centre for Tropical Medicine and Global Health, Nuffield Department of Medicine, University of Oxford, Oxford, UK; Department of Tropical Hygiene, Faculty of Tropical Medicine, Mahidol University, 420/6 Rajvithi Road, Bangkok, 10400, Bangkok, Thailand

## Abstract

Non-academic partners can be vital in successful public engagement activities on antimicrobial resistance. With collaboration between academic and non-academic partners, we developed and launched an open-access web-based application, the ‘antibiotic footprint calculator’, in both Thai and English. The application focused on a good user experience, addressing antibiotic overuse and its impact, and encouraging immediate action. The application was unveiled in joint public engagement activities. From 1 Nov 2021 to 31 July 2022 (9 month period), 2554 players estimated their personal antibiotic footprint by using the application.

## Background

Many organizations have been developing and delivering events and activities aiming to raise awareness of antimicrobial resistance.^1–3^ However, the process and evaluation of these events are rarely published.^4^ In 2019, the ‘antibiotic footprint’ (www.antibioticfootprint.net) concept was described as a tool to communicate the magnitude of antibiotic use in humans and animals to the public by showing each country’s total consumption footprint to support a reduction in overuse of antibiotics worldwide.^5^

Personalized information has been shown to increase awareness of climate change.^6^ In addition, instant feedback and social comparison feedback are associated with initial reductions in energy consumption.^7^ Here, we developed, delivered and evaluated a collaborative public engagement activity conducted jointly by academic and non-academic partners, entitled the ‘antibiotic footprint calculator’ (www.antibioticfootprint.net/calculator).

## Methods

In 2021, we developed an open-access, web-based application, the ‘antibiotic footprint calculator’, in Thai and English, using the concept of carbon footprint calculators.^8,9^ This is because antibiotic use in humans varies by age, gender, local culture, individual attitude to taking antibiotics etc.,^10^ and it would be informative in comparing an individual’s unique antibiotic footprint with fellow citizens and other individuals from different countries.^5^ Academic partners included Chulalongkorn University (Drug System Monitoring and Development Center), Mahidol and Oxford Universities [Mahidol Oxford Tropical Medicine Research Unit (MORU)], and the Thailand Ministry of Public Health (Food and Drug Administration). The non-academic partners included Greenpeace Thailand, PunchUp World and World Animal Protection Thailand. From the outset, the multiple partners had detailed discussions about the scope of the application.

**Figure 1.**
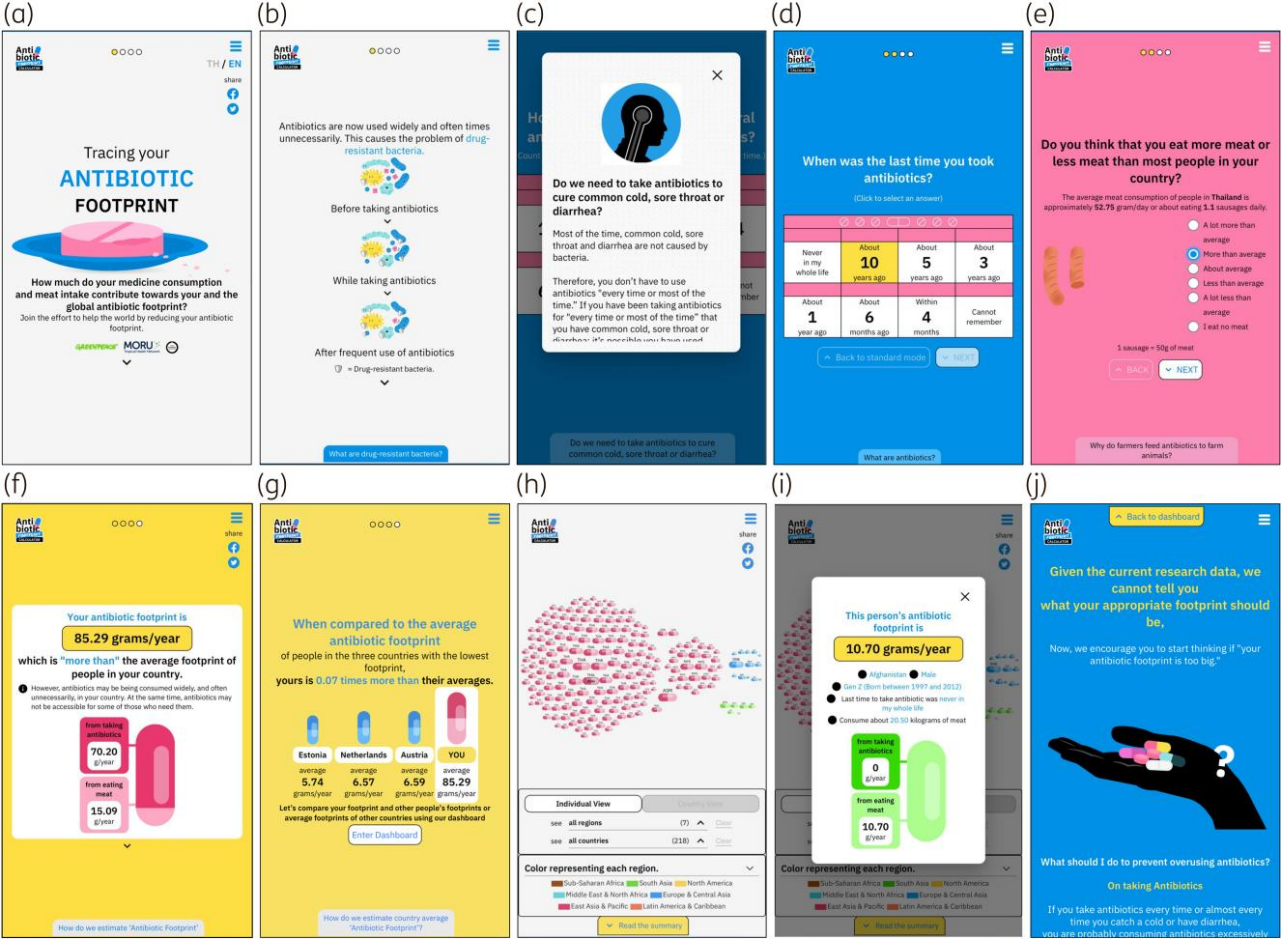
The interface of the antibiotic footprint calculator, showing some pages of the antibiotic footprint calculator when used on mobile phones. (a) A landing page, (b) a simple explanation about antibiotic resistance before playing the quiz, (c) a pop-up additional explanation if players click a note on the page, (d) a question about direct consumption, (e) a question about indirect consumption, (f) result page showing player’s antibiotic footprint, (g) result page showing player’s antibiotic footprint compared with the average antibiotic footprint of countries with low antibiotic footprints, (h) dashboard showing antibiotic footprints of the latest 100 players, (i) antibiotic footprints of other players shown by clicking tablet symbols on the dashboard and (j) conclusion page. This figure appears in colour in the online version of *JAC* and in black and white in the print version of *JAC*.

The new application focused on good user experience, addressing antibiotic overuse and its impact, and encouraging immediate action (Figure 1). We followed evidence-based principles on how to frame antimicrobial resistance as recommended by the Wellcome Trust.^1^ Working with non-academic partners proved beneficial as they cross-checked and commented at all development and piloting stages on whether the application was simple, straightforward and non-technical. Our non-academic partners also had extensive experience in public engagement in other fields that became an asset in designing the application and related activities. To avoid diluting the message, we focused on addressing the overuse of antibiotics, and encouraging individual actions to potentially reduce the overuse of both direct and indirect consumption of antibiotics. Indirect consumption is defined as antibiotic consumption via animals bred for food.^5^

## Description of the antibiotic footprint calculator

The antibiotic footprint calculator (www.antibioticfootprint.net/calculator) calculates both direct and indirect antibiotic consumption. Users, or ‘players’, can decide to give answers to simplified questions (standard mode, which gives less precise results) or more detailed questions (advanced mode, which gives more precise results). Direct consumption is estimated by multiplying the total number of days that a player consumed antibiotics in the past 12 months with the number of grams of antibiotics that an average person consumes per day when taking a course of antibiotics in that particular country. In the standard mode, indirect consumption is estimated by asking players to compare their consumption with the average meat consumption of individuals in their country. In the advanced mode, indirect consumption is estimated by multiplying the kilograms of meat consumed by a player in the past 12 months with the milligrams of antibiotics used per kilogram population correction unit (PCU) of animal meat (i.e. mg/PCU). Consumption of beef, chicken, pork, fish and other seafoods were included in the meat consumption.

Data on total antibiotic consumption per country for humans (in tons and DDD per 1000 population per year) and for animals (in tons and mg/PCU) were from official and open-access data published by each country.^5^ For countries without official and open-access data, we assumed that the parameters of those countries were equal to the average of those parameters from countries with official and open-access data within similar regions, and with the closest income levels. We assumed that indirect consumption would be zero if players did not eat meat or ate only meat labelled with ‘raised without antibiotics’ or ‘no antibiotics ever’. All parameters used were made open access.^11^

Our recommended immediate actions are listed in Table 1. Players can compare their antibiotic footprints with the average antibiotic footprint of countries with low antibiotic footprints and with antibiotic footprints of the latest 100 players. This comparison allowed some level of social comparison feedback.

**Table 1.** Recommended immediate actions for players who completed the application

Think before asking for or buying antibiotics. Every time your doctor is about to prescribe you antibiotics, ask them if there are signs that you really need them. Look for the origins and methods of animal raising before buying meat products. Buy meat from small-scale farmers that use zero or low amounts of antibiotics, ensure that meat is free of antibiotic residues, and, if possible, buy meat products that provide information about the amount of antibiotic used. You can buy industrial meat products labelled ‘No Antibiotics Ever’, ‘Raised Without Antibiotics’ or products from farms that indicate the amount of antibiotics used on their labels.

Ethical permission for this study was obtained from the Ethics Committee of the Faculty of Tropical Medicine, Mahidol University (TMEC 23-002).

## Evaluation and feedback

The application was piloted with the Bangkok Health Research and Ethics Interest Group, a public advisory group based in Thailand, and their inputs were used to improve the content and flow of the application.

The application was officially released on 1 November 2021 with promotional activities during Thailand World Antibiotic Awareness Week. The application was distributed to members of Greenpeace Thailand and World Animal Protection Thailand via e-mail, and also promoted during both partners’ other activities relating to antimicrobial resistance. MORU and other regional headquarters of Greenpeace and World Animal Protection also promoted the application on their websites, and via Twitter and other social media platforms in English.

As of 31 July 2022 (9 month period), based on Google Analytics and anonymous data collection, 3247 people accessed the website, of which 65.7% (*n* = 2134) completed the quiz to receive an estimate of their personal antibiotic footprint. Most players who finished the quiz were from Thailand (*n* = 1376; 64.5%), followed by the UK (*n* = 171; 8.0%) and India (*n* = 114; 5.3%) (Table 2). Of those who completed the quiz, 30.0% (*n* = 640) identified themselves as male, 67.2% (*n* = 1434) as female, 0.7% (*n* = 15) as other and 2.1% (*n* = 45) preferred not to answer. Nearly half of the players (43.6%; *n* = 931) identified themselves as gen Z (born between 1997 and 2012), followed by millennials (27.8%; *n* = 593; born between 1980 and 1996), gen X (19.9%; *n* = 424; born between 1965 and 1979), baby boomers (7.3%; *n* = 156; born between 1946 and 1964), gen alpha (0.8%; *n* = 17, born between 2013 and present) and silent generation (0.2%; *n* = 4; born between 1928 and 1945), while 0.4% (*n* = 9) preferred not to answer. Among countries who had a total number of players more than five, the players from Russia had the highest median antibiotic footprint at 26.1 g/year (*n* = 50 players) and those from Switzerland had the lowest median antibiotic footprint at 0.9 g/year (*n* = 6 players).

**Table 2.** Summary antibiotic footprint of players by countries

Countries	Total number of players^a^	Male gender, % (*n*)	Most common age group, (%; *n*)^b^	Median (IQR, range) antibiotic footprint (g/year)^c^
Thailand	1376	27.3 (375)	Gen Z (51.5; 709)	20.2 (11.8–29.4, 0–112.9)
UK	171	30.4 (52)	Millennials (52.6; 90)	1.0 (0.3–3.1, 0–81.5)
India	114	19.3 (22)	Millennials (51.8; 59)	6.9 (15.4–1.8, 0–99.7)
Spain	99	43.3 (43)	Gen Z (87.9; 87)	9.7 (6.3–15.4, 0–161.5)
Malaysia	87	18.4 (16)	Gen Z (95.4; 83)	23.2 (15.8–31.6, 2.3–101.8)
Russia	50	44 (22)	Millennials (64; 32)	26.1 (18.5–33.7, 7.0–80.2)
USA	27	66.7 (18)	Baby boomers (33.3; 9)	10.9 (4.8–21.8, 0–46.9)
Australia	16	31.3 (5)	Millennials (56.3; 9)	6.5 (3.9–9.4, 0–30.4)
Afghanistan	14	50 (7)	Gen Z (42.9; 6)	10.7 (1.8–23.8, 0–60.5)
Canada	11	72.7 (8)	Baby boomers (45.5; 5)	3.9 (1.3–7.7, 0–24.2)
China	9	22.2 (2)	Millennials (66.7; 6)	25.1 (16.2–29.2, 6.3–42.6)
France	9	44.4 (4)	Gen X (55.6; 5)	1.8 (1.1–3.6, 0–21.4)
Ireland	9	22.2 (2)	Millennials (66.7; 6)	8.8 (0.8–10.1, 0–14.9)
Israel	9	55.6 (5)	Millennials (77.8; 7)	5.9 (1.0–15.6, 0–31.3)
Albania	8	25 (2)	Gen alpha (50; 4)	24.7 (16.3–40.7, 0–56.7)
Belgium	7	56.1 (4)	Millennials (71.4; 5)	3.2 (2.5–12.2, 0.5–35.3)
Switzerland	6	66.7 (4)	Gen X (50; 3)	0.9 (0.5–2.1, 0.4–2.1)
Germany	6	83.3 (5)	Millennials (66.7; 4)	2.4 (1.2–3.5, 0–7.1)
South Africa	6	16.7 (1)	Millennials (83.3; 5)	12.7 (8.1–16.2, 0–17.9)
Others^d^	100	43.0 (43)	Millennials (49.0; 49)	11.1 (3.9–21.5, 0–95.8)
Total	2134	30.0 (640)	Gen Z (43.6; 931)	16.2 (7.5–26.7, 0–161.5)

Total number of players who completed the quiz. Countries that had the total number of players more than five are listed.

Gen alpha was defined as those born between 2013 and present, gen Z was defined as those who born between 1997 and 2012, millennials were defined as those born between 1980 and 1996, gen X was defined as those born between 1965 and 1979, baby boomers were defined as those born between 1946 and 1964, and the silent generation was defined as those born between 1928 and 1945.

Antibiotic footprint could be 0 for players who answered that they had not consumed any antibiotics directly and did not consume any meat (or always consumed only meat labelled ‘raised without antibiotics’ or ‘no antibiotics ever’) during the last 12 months.

Other countries and economies include American Samoa, Italy, the Netherlands and Vietnam (*n* = 5), Andorra, Cambodia, Denmark, New Zealand, the Philippines and Turkey (*n* = 4), Algeria, Argentina, Armenia, Belarus, Brazil, Greece, Hong Kong, Japan, Kenya, Laos, Norway, Singapore, Slovenia, South Korea, Sweden, Taiwan and Uzbekistan (*n* = 2), Austria, Bahrain, Benin, Belize, Brunei, Cameroon, Colombia, Cayman Islands, Czech Republic, Gibraltar, Indonesia, Iraq, Kazakhstan, Lebanon, Lithuania, Nigeria, Nepal, Pakistan, Saudi Arabia, Ukraine, United Arab of Emirates and Uruguay (*n* = 1).

At the end of the quiz, players were provided with questionnaires for feedback in Thai and English, including eight questions with a 5-point Likert-scale and one open-ended question for comments, advice or suggestions. Only 32 players provided feedback. For the question, how positive or negative they felt about the antibiotic footprint calculator application, all answered either ‘highly positive’ or ‘positive’.

## Discussion

It is important to raise awareness of the problem of the overuse of antibiotics, and over-the-counter availability of antibiotics in many low- and middle-income countries (LMICs),^12^ as well as the use of antibiotics in animals bred for food. People should act immediately to reduce their direct and indirect consumption of antibiotics, as recommended in Table 1. Professional organizations should also consider adding the web addresses of antibiotic footprint calculators onto their websites.

The application has a number of limitations. Some players might play more than once or not answer accurately. It is possible that some players were just testing the application (e.g. those who answered that they were from Afghanistan and were gen alpha (*n* = 5), as those were the first options in the drop-down menus) and their answers may not have been accurate. A self-reporting bias in meat consumption is also common.^13,14^ The availability of open-access data on national antibiotic consumption in human and animal sectors is still limited.^5^ Similar to carbon footprint calculators,^8,9^ formulas to estimate individual antibiotic footprints could be improved based on data availability and future studies. Statistics on the estimated antibiotic footprint of players cannot be directly extrapolated to the general population.^15^ Although more than 2500 players completed the quiz on the application, evaluation of its impact is still limited. It is likely that the addition of more local languages and local partners could improve its engagement with other non-native English-speaking countries. Further development, evaluation and studies on antibiotic footprint calculators (e.g. large studies using approaches similar to those conducted for carbon footprint calculators^6–9^) are required.

## Conclusions

Overall, we would like to highlight potential benefits of public engagement activities with collaboration between multiple academic and non-academic partners. Diverse partners bring different sets of expertise and networks and tremendously improve engagement activities or events related to antimicrobial resistance.
